# Understanding the Energy Band Mechanism in MoS_2_/Co_3_O_4_ Heterojunction-Based Bioplastics Affected by Carrier Concentration

**DOI:** 10.3390/nano15040297

**Published:** 2025-02-15

**Authors:** Posak Tippo, Wattikon Sroila

**Affiliations:** 1 Office of Research Administration, Chiang Mai University, Chiang Mai 50200, Thailand; 2 Department of Physics and Materials Science, Faculty of Science, Chiang Mai University, Chiang Mai 50200, Thailand

**Keywords:** MoS_2_, Co_3_O_4_, bioplastics, energy band mechanism, heterojunction

## Abstract

Bioplastics are adopted to replace fossil-based plastics because they are microplastic-free and self-degradable without releasing greenhouse gasses. Despite having many benefits, the main applications of bioplastics are packaging and kitchenware. Moreover, the utilization of bioplastics in electronic applications is still underexplored. Consequently, the development of bioplastics for electronic applications, especially heterojunctions, is essential. Here, we report a novel molybdenum disulfide (MoS_2_)/cobalt oxide (Co_3_O_4_) heterojunction based on bioplastic semiconductors, with agar as a matrix. This work also exposes the effect of carrier concentration on the mechanism of an energy band. Using the density of state in three dimensions, Anderson’s rule, and the Fermi energy level calculated by carrier concentration, we find that the energy gaps of the MoS_2_/Co_3_O_4_ heterojunction at various concentrations almost match the energy gap evaluated by Tauc’s relation. Additionally, leveraging the MoS_2_/Co_3_O_4_ heterojunction as a photodetector, the optimized device indicates an ideality factor of 1.59, a response time of 127 ms, and a recovery time of 115 ms. Our work not only represents a significant step towards using bioplastics in electronic applications but also reveals the mechanism of the energy band affected by carrier concentration.

## 1. Introduction

Our world faces a plastic pollution crisis and global warming caused by fossil-based plastic, which has a low decay rate and causes a high release of carbon dioxide (CO_2_) and toxic gas due to its production and incineration processes [1,2,3,4]. Therefore, alternative materials to replace fossil-based plastic are urgent. Bioplastics tend to be adopted to replace fossil-based plastics because they are self-degradable and microplastic-free [5,6]. Furthermore, growing crops to produce bioplastics not only increases their employment but also reduces the CO_2_ from photosynthesis [7,8]. Although bioplastics have many benefits, their main applications today are packaging and kitchenware [9,10]. By considering the global market value, the market value of electronics is much higher than that of plastic packaging and kitchenware [11,12]. Thus, the development of bioplastics for electronic applications is significant, especially heterojunctions, which are the building blocks for all electronic devices [13,14,15].

Currently, there are numerous studies on heterojunctions for developing solar cells, diodes, transistors, and photodetectors [13,14,15]. To understand and control the characteristics of a heterojunction, the energy band alignment at the interfaces of the semiconductor is required. Generally, Anderson’s rule is widely used to construct the energy band alignment of the heterojunction between two semiconductors, using the vacuum levels as the reference point [16,17]. Nevertheless, many published papers reported that Anderson’s rule cannot accurately predict real band offsets because it only considers vacuum levels without other factors, such as non-existent vacuum separation and carrier concentrations, which significantly impact the energy band [18,19,20]. Consequently, it is essential to study the mechanism of the energy band affected by carrier concentrations and calculate the energy gap of the heterojunction. Moreover, the material required to study this mechanism is necessary as well. It has been reported that heterojunctions consisting of MoS_2_ are promising in photodetector applications [21,22].

This work presents a MoS_2_/Co_3_O_4_ heterojunction based on bioplastic semiconductors and the effect of carrier concentration on the energy band mechanism of the MoS_2_/Co_3_O_4_ heterojunction. The Co_3_O_4_ nanoparticles were synthesized using wet chemical precipitation. Meanwhile, MoS_2_ flakes can be obtained from electrochemical exfoliation. The bioplastic semiconductors were achieved using a gel-casting method, with agar as a matrix and MoS_2_ and Co_3_O_4_ as fillers. To reveal the morphology and crystallinity of MoS_2_ and Co_3_O_4_, a high-resolution transmission electron microscope (HRTEM), selected area diffraction (SAED), and X-ray diffractometer (XRD) were used. The carrier concentration at various MoS_2_ and Co_3_O_4_ content was measured using the Hall effect via the Van der Pauw method. For calculating the Fermi energy level and energy gap of the MoS_2_/Co_3_O_4_ heterojunction, we used the density of state (DOS) in three dimensions, Anderson’s rule, and Tauc’s relation. Additionally, the electrical properties and the characteristics of the MoS_2_/Co_3_O_4_ heterojunction utilized as a photodetector were investigated.

## 2. Experimental

### 2.1. Wet Chemical Precipitation of Co_3_O_4_

In total, 3 g of cobalt nitrate hexahydrate (Co(NO_3_)_2_·6H_2_O, ≥98%, Sigma-Aldrich, Saint Louis, MO, USA) was dissolved in 75 mL of deionized (DI) water at 80 °C. The aqueous sodium hydroxide solution (NaOH, RCI Labscan, Bangkok, Thailand) was then gradually dropped into the cobalt acetate solution until the pH was 14. At this stage, the solution was changed from pink to teal due to the formation of cobalt hydroxide. After precipitation, the particles were filtered and washed with DI water several times until the pH was 7. Subsequently, the particles were dried at 100 °C and decomposed to cobalt oxide (Co_3_O_4_) at 400 °C for 4 h.

### 2.2. Electrochemical Exfoliation of MoS_2_

In total, 3 g of MoS_2_ powders (<2 μm, 98%, Sigma-Aldrich, Missouri, USA) was used as the cathode and placed parallel to platinum foil as the anode with a distance of 3 cm [23]. Both electrodes were then immersed in 0.5 M sodium sulfate (Na_2_SO_4_, RCI Labscan, Bangkok, Thailand) solution, and the electrochemical exfoliation of MoS_2_ was conducted at a voltage of 10 V for 1 h. After that, the MoS_2_ exfoliated flakes were filtered and washed with DI water several times to remove the Na_2_SO_4_. Finally, the flakes were dried overnight at 60 °C.

### 2.3. Fabrication of MoS_2_/Co_3_O_4_ Heterojunction Based on Bioplastic Semiconductor

**Figure 1:**
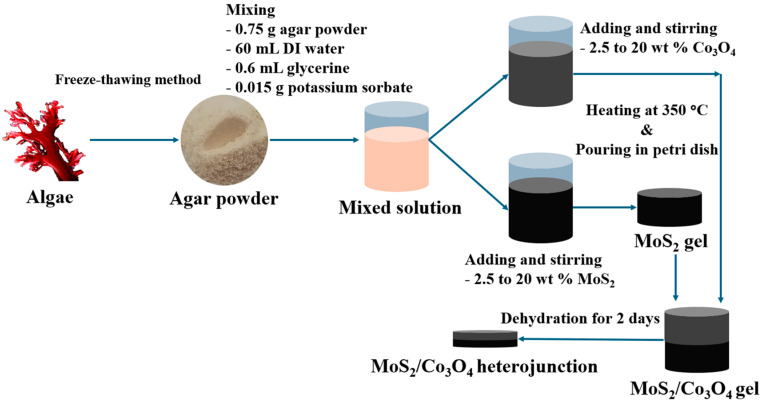
The schematic diagram for the fabrication of the MoS_2_/Co_3_O_4_ heterojunction.

The agar powder was used as a matrix of bioplastic semiconductors. This powder was extracted from seaweed using a freeze–thawing method, as reported in our previous work [24]. In total, 0.75 g agar powder was immersed in 60 mL DI water for 10 min. Then, 0.6 mL glycerine, 0.015 g potassium sorbate, and MoS_2_ flakes with concentrations of 2.5, 5, 10, and 20 wt% for each condition were added. After that, the mixed solution was heated at 350 °C until boiling and stopped immediately. Subsequently, the solution containing MoS_2_ flakes was poured into the Petri dish and left at room temperature for 5 min or until gelation. In the meantime, the solution for making Co_3_O_4_ gel was prepared using the same method and conditions as MoS_2_ gel. After finishing the preparation, the solution containing Co_3_O_4_ nanoparticles was poured on top of MoS_2_ gel and left at room temperature until forming the Co_3_O_4_ gel. Finally, the MoS_2_/Co_3_O_4_ heterojunction gel was dehydrated to form the bioplastic semiconductor films by being kept in a silica gel container for 2 days. It is noted that the relationship between gel and film thickness can be obtained from our previous work [24]. The schematic diagram of the fabrication is displayed in Figure 1.

### 2.4. Characterization

In this study, the instruments and the methods for characterization consist of a high-resolution transmission electron microscope (HRTEM, JEOL JEM-2010, Tokyo, Japan), selected area diffraction (SAED), an X-ray diffractometer (XRD, RIGAKU MiniFlex II, Tokyo, Japan), the Hall effect via the Van der Pauw (VDP) method, a UV–visible spectrophotometer (Varian Cary 50, Sydney, Australia), the density of state (DOS) in 3 dimensions, Tauc’s relation, and a source measure unit (SMU, Keithley 2450, Miamisburg, OH, USA). 

## 3. Results and Discussion

### 3.1. Morphology and Crystallinity

**Figure 2:**
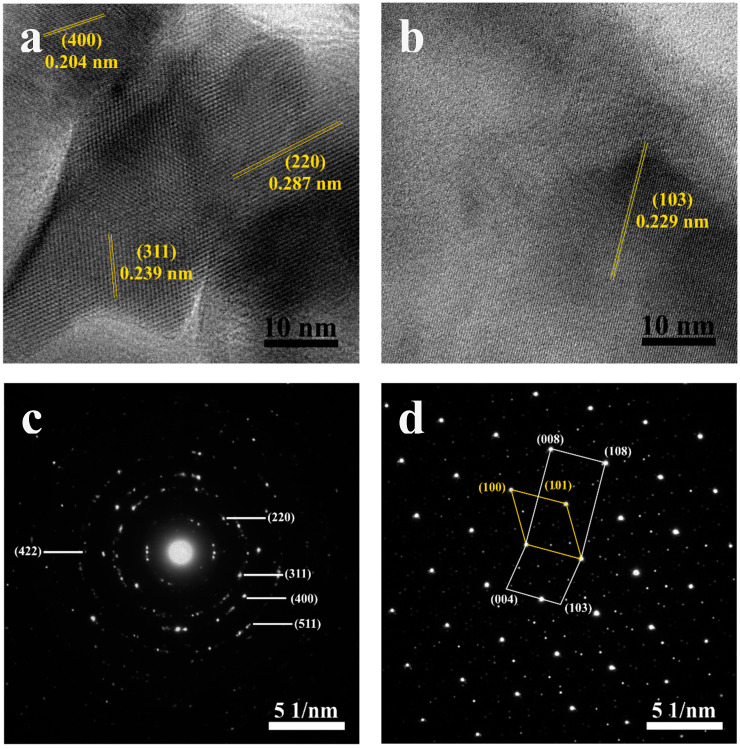
HRTEM images of (**a**) Co_3_O_4_ and (**b**) MoS_2_. SAED patterns of (**c**) Co_3_O_4_ and (**d**) MoS_2_.

The HRTEM images of Co_3_O_4_ and MoS_2_ are shown in Figure 2a,b. We found that the lattice fringe spacing of Co_3_O_4_ is 0.287, 0.239, and 0.204 nm, which are associated with the lattice plane of (220), (311), and (400), respectively. These results match JCPDS No. 00-042-1467 well. Simultaneously, the MoS_2_ only illustrates the lattice fringe spacing of 0.229 nm related to the (103) plane of JCPDS No. 06-0097. To further investigate the insight crystallinity of Co_3_O_4_ and MoS_2_, SAED patterns were used, as displayed in Figure 2c,d. The SAED patterns of Co_3_O_4_ in Figure 2c illustrate a ring pattern, reflecting the crystalline structure, while MoS_2_ in Figure 2d displays a symmetry pattern, indicating high crystallinity. The additional results, including the SEM images of the cross-section, the thickness, and elemental confirmation of MoS_2_/Co_3_O_4_ heterojunctions, are shown in Figures S1 and S2 of the Supplementary Information.

**Figure 3:**
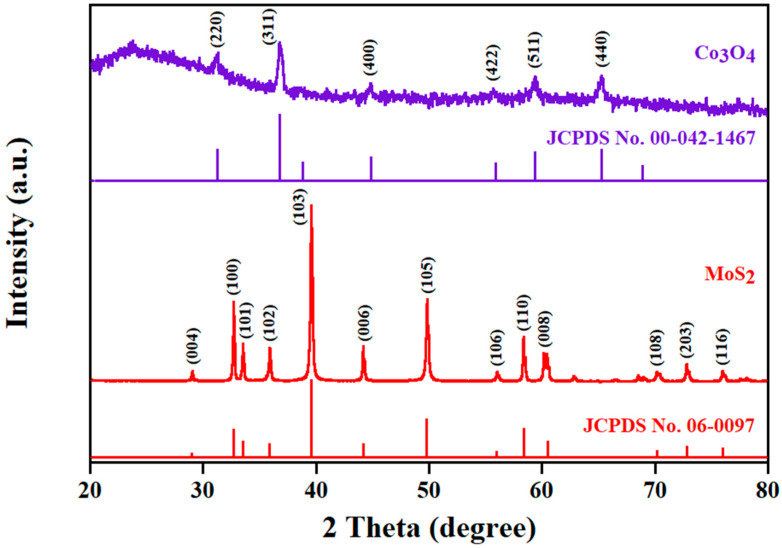
XRD patterns of Co_3_O_4_ and MoS_2_.

To confirm the HRTEM and SAED results, we used XRD patterns, as shown in Figure 3. It was found that the patterns of Co_3_O_4_ and MoS_2_ are in agreement with the JCPDS and SAED results. The highest peak of Co_3_O_4_ is (311), located at 36.76°. For MoS_2_, the location of the highest peak is 39.58°, consistent with (103). Based on this information, Bragg and Scherrer equations can be used to reveal the d-spacing (*d*), crystallite size (*D*), and dislocation density (*δ*) as follows [24,25]:

(1) 2dsinθ=nλ

(2) $$D=\frac{0.9\lambda }{\beta \cos\theta }$$

(3) $$\delta =\frac{1}{D^{2}}$$

where *θ* is the Bragg angle, *n* is the diffraction order, *λ is* the wavelength of the X-ray (0.15418 nm), and *β* is the full width at half maximum (FWHM) of the diffraction peak. By calculating values from Equation (1), the d-spacing of the highest peak is 0.244 nm for Co_3_O_4_ and 0.228 nm for MoS_2_. These values match the lattice fringe spacing from the HRTEM well. The crystallite sizes of Co_3_O_4_ and MoS_2_ derived from Equation (2) are 17.49 nm and 42.46 nm. Meanwhile, the dislocation densities calculated using Equation (3) are 3.27 × 10^15^ lines m^−2^ for Co_3_O_4_ and 5.55 × 10^14^ lines m^−2^ for MoS_2_. The lower values of the dislocation densities reflect higher crystallinity, which agrees with the SAED results well.

### 3.2. Electrical Properties and Mechanism of Energy Band

**Figure 4:**
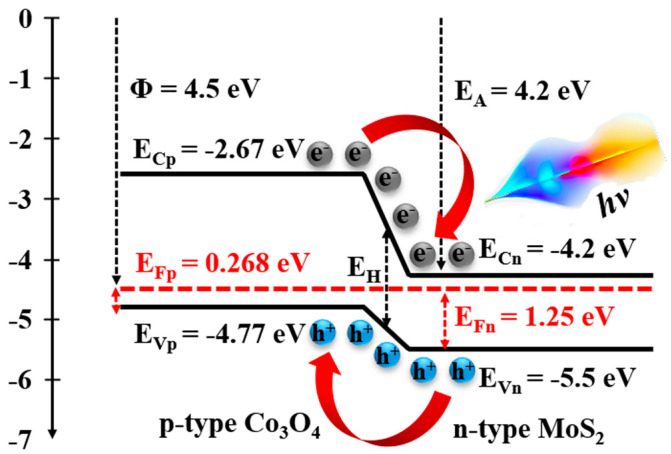
The energy band of the 2.5 wt% MoS_2_/Co_3_O_4_ heterojunction. It is noted that the position of *E_Fp_* and *E_Fn_* in the vacuum level is slightly different, caused by the fragility of Anderson’s rule as described in Equations (9) and (10). For more details on the charge transport mechanism, see Supplementary Section S2.

To reveal the type of semiconductor and the electrical properties of individual Co_3_O_4_ and MoS_2_ bioplastics, we use the Hall effect via the Van der Pauw method. See Supplementary Section S1 for details. The values of the Hall coefficient, resistance, carrier concentration, and semiconductor type can be found in Table 1. By increasing the concentration of Co_3_O_4_ and MoS_2_ from 2.5 to 5 wt%, the carrier concentrations of both materials are promoted. However, increasing the concentration too high (>5 wt%) reduces the carrier concentrations. This decrease is caused by trap-assisted recombination, as excess filler induces a deep trap state in the bioplastic semiconductor during Hall measurements [26,27]. By considering the Hall coefficient, the individual Co_3_O_4_ and MoS_2_ bioplastics at various concentrations indicate similar types of semiconductors to the general reports [28,29]. To understand the mechanism of the energy band affected by the carrier concentration, we require the Fermi energy level and energy gap of Co_3_O_4_ and MoS_2_, which can be calculated using the following equation [14,30,31]:

(4) $$E_{Fn}-E_{Fi}=kT\ln\frac{n_{0}+\delta n}{n_{i}}$$

(5) $$E_{Fi}-E_{Fp}=kT\ln\frac{p_{0}+\delta p}{n_{i}}$$

where *E_Fn_* is the Fermi energy levels of electrons, *E_Fi_* is the intrinsic Fermi energy, *E_Fp_* is the Fermi energy levels of holes, *k* is the Boltzmann constant, *T* is the temperature (°K), *n_0_* is the carrier concentration of an n-type semiconductor, *δn* = *δp* is the excess carrier concentrations, *p_0_* is the carrier concentration of a p-type semiconductor, and *n_i_* is the intrinsic carrier concentration. For the calculation of the intrinsic carrier concentration, we use the equation of the DOS in three dimensions as follows [32,33]:

(6) $$n_{i}=2{\left(\frac{kT}{2\pi \hbar^{2}}\right)}^{3/2}{\left(m_{e}m_{h}\right)}^{3/4}\exp(\frac{-E_{g}}{2kT})$$

where *m_e_* is the mass of the electron, *m_h_* is the mass of the hole, *E_g_* is the energy gap, and *ħ* is reduced Planck’s constant. It is noted that the *E_g_* of Co_3_O_4_ = 2.1 eV [28,34], MoS_2_ = 1.3 eV [29,35], and *m_e_* = *m_h_*. The Fermi energy levels of Co_3_O_4_ and MoS_2_ calculated using Equations (4)–(6) are illustrated in Table 1. Based on the work function (Φ) of 4.5 eV for Co_3_O_4_ [36,37] and the electron affinity of 4.2 eV for MoS_2_ [29,38] estimated by previous reports, the energy band of the MoS_2_/Co_3_O_4_ heterojunction can be generated using Anderson’s rule [16,17,39]:

(7) $$E_{V}=\Phi +E_{F}$$

(8) $$E_{C}=E_{A}=E_{V}-E_{g}$$

where *E_V_* is the valence band, *E_A_* is the electron affinity, and *E_C_* is the conduction band. By deriving results from Equations (7) and (8), we have the energy band of the MoS_2_/Co_3_O_4_ heterojunction, as displayed in Figure 4. We found that the energy gap of the MoS_2_/Co_3_O_4_ heterojunction (*E_H_*) is similar to the conduction band offsets (*ΔE_C_*). Therefore, *E_H_* can be revealed by [14,17]

(9) $$E_{H}=E_{Cp}-E_{Cn}$$

where *E_Cp_* is the energy level of the conduction band for a p-type semiconductor and *E_Cn_* is the energy level of the conduction band for an n-type semiconductor. It is well known that Anderson’s rule usually fails to predict real band offsets because the heterojunction is filled with solids, resulting in non-existent vacuum separation [18,19,20]. To eliminate this vacuum, we apply absolute value to Equation (9). By the modification of Equation (9) to estimate the *E_H_* affected by the relative change in the Fermi energy levels (*ΔE_F_*) associated with carrier concentration, we obtain

(10) $$E_{H}=\left|\left(\left|E_{Cp}\right|+\Delta E_{Fp})-(\left|E_{Cn}\right|+\Delta E_{Fn}\right)\right|$$

By using Equations (9) and (10), the *E_H_* of 2.5 to 20 wt% MoS_2_/Co_3_O_4_ heterojunctions is found to be 1.53, 1.50, 1.58, and 1.63 eV, respectively. To confirm these calculated values, Tauc’s relation is used, which will be discussed with regard to the optical properties.

### 3.3. Optical Properties

**Figure 5:**
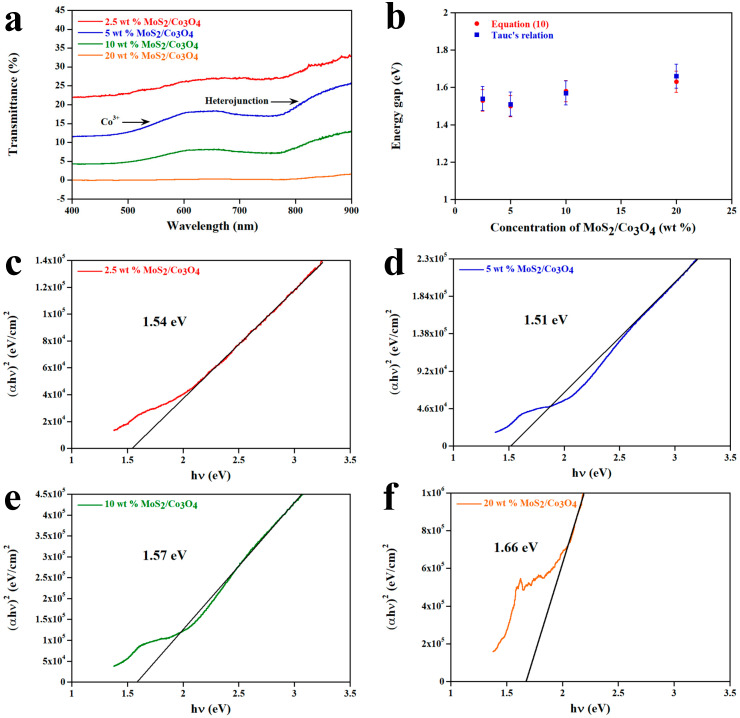
(**a**) The transmittance of the MoS_2_/Co_3_O_4_ heterojunction at various concentrations. (**b**) The energy gaps from Tauc’s relation and Equation (10) versus the concentration of MoS_2_/Co_3_O_4_. Tauc plot of (**c**) 2.5 wt%, (**d**) 5 wt%, (**e**) 10 wt%, and (**f**) 20 wt% MoS_2_/Co_3_O_4_ heterojunctions.

Figure 5a displays the transmittance of the MoS_2_/Co_3_O_4_ heterojunction at various concentrations. The average transmittance (wavelength of 400–700 nm) decreases from 24.57 to 0.14% when the concentration increases from 2.5 to 20 wt%. Additionally, it was found that the MoS_2_/Co_3_O_4_ heterojunction absorbs two ranges of wavelengths. The first absorption occurs at the wavelength from 770 to 850 nm caused by the heterojunction. Another absorption originates at the wavelength from 500 to 600 nm. This absorption is associated with Co^3+^, reflecting a natural characteristic of Co_3_O_4_ [40]. In order to identify the energy gap of the MoS_2_/Co_3_O_4_ heterojunction, we use Tauc’s relation, which is calculated using the equations as follows [24,41]:

(11) $$A_{b}=2-\log(\%T)$$

(12) $$\alpha =2.303(\frac{A_{b}}{t})$$

(13) $${\left(\alpha h\nu \right)}^{1/n}=A(h\nu -E_{g})$$

where *A_b_* is the absorbance, *%T* is the percentage of transmittance, *α* is the absorption coefficient, *t* is the thickness of the film, *v* is the photon frequency, *h* is the Planck constant, *A* is the proportionality constant, and *n* is equal to 1/2 or 2 for direct and indirect transitions, respectively. By plotting the *hv* versus (*αhv*)^1/n^, as shown in Figure 5c–f, the energy gaps of 2.5 to 20 wt% MoS_2_/Co_3_O_4_ heterojunctions are 1.54, 1.51, 1.57, and 1.66 eV, respectively. These values almost match the values of the energy gaps calculated from Equation (10). To make this easier to observe, we plot the values of energy gaps from Tauc’s relation and Equation (10) versus the concentration of the MoS_2_/Co_3_O_4_ heterojunction, as illustrated in Figure 5b.

### 3.4. Characteristics of Photodetector

**Figure 6:**
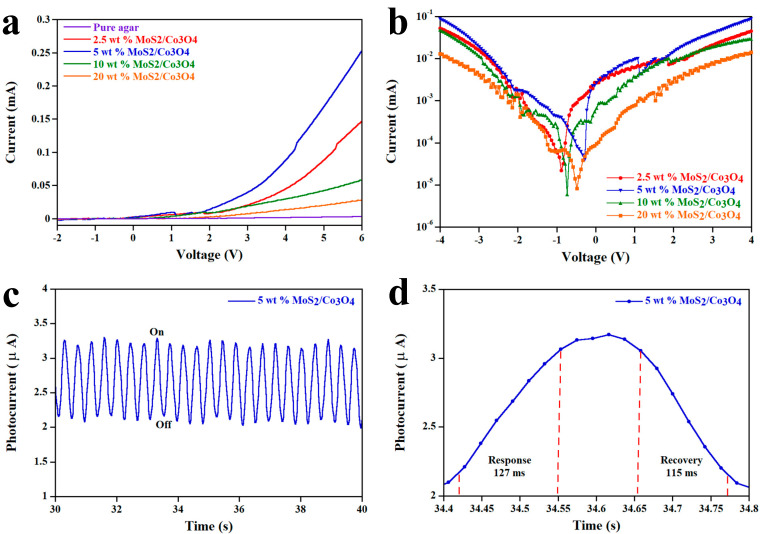
The characteristics of the MoS_2_/Co_3_O_4_ photodetectors consist of (**a**) I–V curves, (**b**) semi-log plots, (**c**) the 5 wt% MoS_2_/Co_3_O_4_ photodetector under the light on/off of 2.5 Hz, and (**d**) the response and recovery times of the 5 wt% MoS_2_/Co_3_O_4_ photodetector evaluated between 10% and 90% of the maximum photocurrent.

The current–voltage (I–V) characteristics of the MoS_2_/Co_3_O_4_ heterojunction at various concentrations are shown in Figure 6a. It is noted that the setup information for the electronic measurement and optical image of the device is demonstrated in Figure S8. We found that the I–V curves are not linear, indicating a characteristic of the heterojunction. Furthermore, the 5 wt% MoS_2_/Co_3_O_4_ heterojunction displays the highest forward current followed by 2.5, 10, and 20 wt%, respectively, caused by the energy gap values. The lower the energy gap, the lower the energy or potential required to excite electrons from the valence band to the conduction band [42]. Thus, the results of the I-V characteristics support and are in agreement with Tauc’s relation and Equation (10). The confirmation of the Ohmic contact between the electrodes and the matrix containing MoS_2_ and Co_3_O_4_ is displayed in Figure S3. It is noted that the I–V curve of pure agar is shown in Figure S7. To reveal the ideality factor (*η*) of the MoS_2_/Co_3_O_4_ heterojunctions, we use semi-log plots, as illustrated in Figure 6b and the equation as follows [43]:

(14) $$\ln(I)=(\frac{q}{\eta \mathrm{kT}})V+\ln(I_{0})$$

where *I*_0_ is the dark saturation current, *k* is Boltzmann’s constant, *V* is the change in voltage, *T* is the temperature, *q* is the elementary charge, and *I* is the change in current. Using Equation (14), the ideality factors of the 2.5 to 20 wt% MoS_2_/Co_3_O_4_ heterojunctions are found to be 2.94, 1.59, 3.32, and 4.89, respectively.

It can be interpreted that the *η* between 1 and 2 (the value for a standard photodiode) originates from major recombination limited by minority carriers and minor recombination limited by majority carriers [15]. For the *η* higher than 2, the recombination of the carriers occurs at the interface of the p-n junction caused by trap-assisted recombination [15]. Therefore, the heterojunction with a higher *η* indicates a lower forward current than the heterojunction with a lower *η*, comparing at the same potential. As mentioned above, the forward current is associated with the energy gap affected by carrier concentration, which is directly related to the filler concentration. As a result, the *η* of MoS_2_/Co_3_O_4_ heterojunctions can be optimized by varying the concentration of MoS_2_ and Co_3_O_4_. By considering the energy gap, I–V curves, ideality factor, and carrier concentration of the individual MoS_2_ and Co_3_O_4_, we determine that the 5 wt% MoS_2_/Co_3_O_4_ heterojunction is an optimized condition for the photodetector. In order to investigate the response and recovery times, the 5 wt% MoS_2_/Co_3_O_4_ photodetector is exposed to light with an on/off frequency of 2.5 Hz, as shown in Figure 6c. It was observed that the device responds well to light. To further evaluate the values of the response and recovery times, Figure 6d is used. We found that the response and recovery times at zero bias of the 5 wt% MoS_2_/Co_3_O_4_ photodetector are 127 ms and 115 ms, respectively. From the different values between the response and recovery times, it can be interpreted that the rate of electron–hole pair generation by absorbing light is lower than that of the recombination rate. This is caused by trap-assisted recombination, which reduces the generation of electron–hole pairs under absorbing light and promotes electron–hole pair recombination while not absorbing light [15,26,27]. As displayed in Figure S4, a 5 wt% MoS_2_/Co_3_O_4_ photodetector indicates an open-circuit voltage (V_OC_) of 104 mV, a short-circuit current density (J_SC_) of 1.01 μA, and a fill factor (FF) of 0.54, which may have potential in photovoltaic applications. The stability of 5 wt% MoS_2_/Co_3_O_4_ is illustrated in Figure S5. To identify the figure of merit of the 5 wt% MoS_2_/Co_3_O_4_ photodetector, we use responsivity (*R*), specific detectivity (*D*), and noise equivalent power (*NEP*). These factors can be calculated using the equations as follows [21]:

(15) $$R=\frac{I_{ph}}{P_{d}A_{O}}$$

(16) $$NEP=\frac{\sqrt{2eI_{d}}}{R}$$

(17) $$D=\frac{R\sqrt{A_{O}}}{NEP}$$

where *I_ph_* = *I_i_ − I_d_* is the net photocurrent, *I_i_* is the light current, *I_d_* is the dark current, *P_d_* is the power density of incident light, *A_O_* is the active area of the photodetector, and e is the elementary charge. By calculating values from Equations (15)–(17), the values of *R*, *NEP*, and *D* are 50 μA W^−1^, 1.6 × 10^−8^ W Hz^−1/2^, and 1.56 × 10^7^ Jones, respectively. The comparisons of the 5 wt% MoS_2_/Co_3_O_4_ photodetector based on bioplastic semiconductors to other photodetectors based on ceramic semiconductors are shown in Table 2.

## 4. Conclusions

In this work, a MoS_2_/Co_3_O_4_ heterojunction based on bioplastic semiconductors was successfully fabricated by the gel-casting method, with agar as a matrix and MoS_2_ and Co_3_O_4_ as fillers. The agar, MoS_2_, and Co_3_O_4_ were synthesized using freeze–thawing, electrochemical exfoliation, and wet chemical precipitation methods, respectively. SAED and XRD patterns match well and are also consistent with the diffraction standards. Moreover, lattice fringe spacing from the HRTEM image agrees with the d-spacing of XRD. By increasing the concentration of MoS_2_ and Co_3_O_4_, the carrier concentrations in both bioplastic semiconductors are changed. In order to reveal the effect of the carrier concentrations on the mechanism of the energy band and the energy gap values, we use the DOS, Fermi energy level, Anderson’s rule, and Equation (10). Our findings indicate that the carrier concentrations significantly change the energy band of the MoS_2_/Co_3_O_4_ heterojunction. Furthermore, the energy gap values of the MoS_2_/Co_3_O_4_ heterojunction calculated using Equation (10) almost match the energy gaps received from Tauc’s relation. By utilizing the MoS_2_/Co_3_O_4_ heterojunction as a photodetector, we found that the 5 wt% is an optimized condition, indicating an ideality factor of 1.59, a response time of 127 ms, and a recovery time of 115 ms. This work not only helps to understand the mechanism of the energy band in the heterojunction but also opens pathways for developing other bioplastics with different fillers and matrices, potentially transforming future approaches to photodetector design and other electronic applications.

## Figures and Tables

**Table 1:** The Hall coefficient, resistance, carrier concentration, type of semiconductor, and Fermi energy levels of the individual MoS_2_ and Co_3_O_4_ at various concentrations.

Bioplastic Semiconductors	Hall Coefficient	Resistance (MΩ)	Carrier Concentration (cm^−3^)	Type	*E_F_* (eV)
2.5 wt% MoS_2_	−504	2.9	−1.24 × 10^16^	N	1.250
5 wt% MoS_2_	−205	1.8	−3.04 × 10^16^	N	1.274
10 wt% MoS_2_	−3548	4.6	−1.76 × 10^15^	N	1.199
20 wt% MoS_2_	−7880	12	−7.92 × 10^14^	N	1.178
2.5 wt% Co_3_O_4_	1044	2.2	5.98 × 10^15^	P	0.268
5 wt% Co_3_O_4_	694	1.9	8.99 × 10^15^	P	0.258
10 wt% Co_3_O_4_	1135	5.5	5.49 × 10^15^	P	0.271
20 wt% Co_3_O_4_	2530	8.6	2.47 × 10^15^	P	0.292

**Table 2:** The performance of the 5 wt% MoS_2_/Co_3_O_4_ photodetector based on bioplastic semiconductors compared to other photodetectors based on ceramic semiconductors.

Methods	Materials	Ideality Factors	Response Time (ms)	Recovery Time (ms)	References
Precipitation and gel-casting	MoS_2_/Co_3_O_4_	1.59	127	115	This work
Molecular beam epitaxy	MoS_2_/GaN	-	33	31	[21]
Thermal evaporator and thermal oxidation	NiO/n-Si	25	1,700	850	[44]
Direct vapor technique	n-SnSe_2_/p-Si	2.17	701	689	[45]
Heat treatment	SrTiO_3_	-	1140	1190	[46]
DC magnetron sputtering	n-ZnO/p-NiO	2.59	197	537	[47]
Microwave-assisted synthesis	Ni-WS_2_/Si	1.1	63	100	[48]

## Data Availability

All data supporting the findings of this study are provided in the article, as well as in the Supplementary Information.

## Supplementary Materials

The following supporting information can be downloaded at: https://www.mdpi.com/article/10.3390/nano15040297/s1, Figure S1: The SEM images of cross-sections (a) 2.5 wt %, (b) 5 wt %, (c) 10 wt %, and (d) 20 wt % MoS_2_/Co_3_O_4_. Figure S2: The EDS confirms elements of MoS_2_/Co_3_O_4_ heterojunction. Figure S3: I-V curves of Co_3_O_4_/Cu and MoS_2_/ITO. Figure S4: J-V curve of MoS_2_/Co_3_O_4_ photodetector under the illumination of 1000 W/m^2^. Figure S5: The stability of 5 wt % MoS_2_/Co_3_O_4_ under light on/off 2.5 Hz for 35 mins. Figure S6: Diagram of Hall measurement via the van der Pauw method [49]. Figure S7: I-V curve of pure agar. Figure S8: The setup information for the electronic measurement and the optical image of MoS_2_/Co_3_O_4_ heterojunction as a photodetector. References [13,14,15,49] are cited in the supplementary materials.
