# Supplementary material for: Understanding the Energy Band Mechanism in MoS2/Co3O4 Heterojunction-Based Bioplastics Affected by Carrier Concentration
Source: Nanomaterials (Basel). 2025 Feb 15;15(4):297. doi: 10.3390/nano15040297 (PMC11858651; doi:10.3390/nano15040297)
Supplement: Supplementary file 1 [file nanomaterials-15-00297-s001.zip › nanomaterials-3468739-supplementary.pdf]

Supplementary information to

Understanding the Energy Band Mechanism in MoS_2/Co_3O_4 Heterojunction-Based Bioplastics Affected by Carrier Concentration

Posak Tippo [1,2,*] and Wattikon Sroila [2]

[1] Office of Research Administration, Chiang Mai University, Chiang Mai 50200, Thailand
[2] Department of Physics and Materials Science, Faculty of Science, Chiang Mai University, Chiang Mai 50200, Thailand
* Correspondence: posaktippo@gmail.com

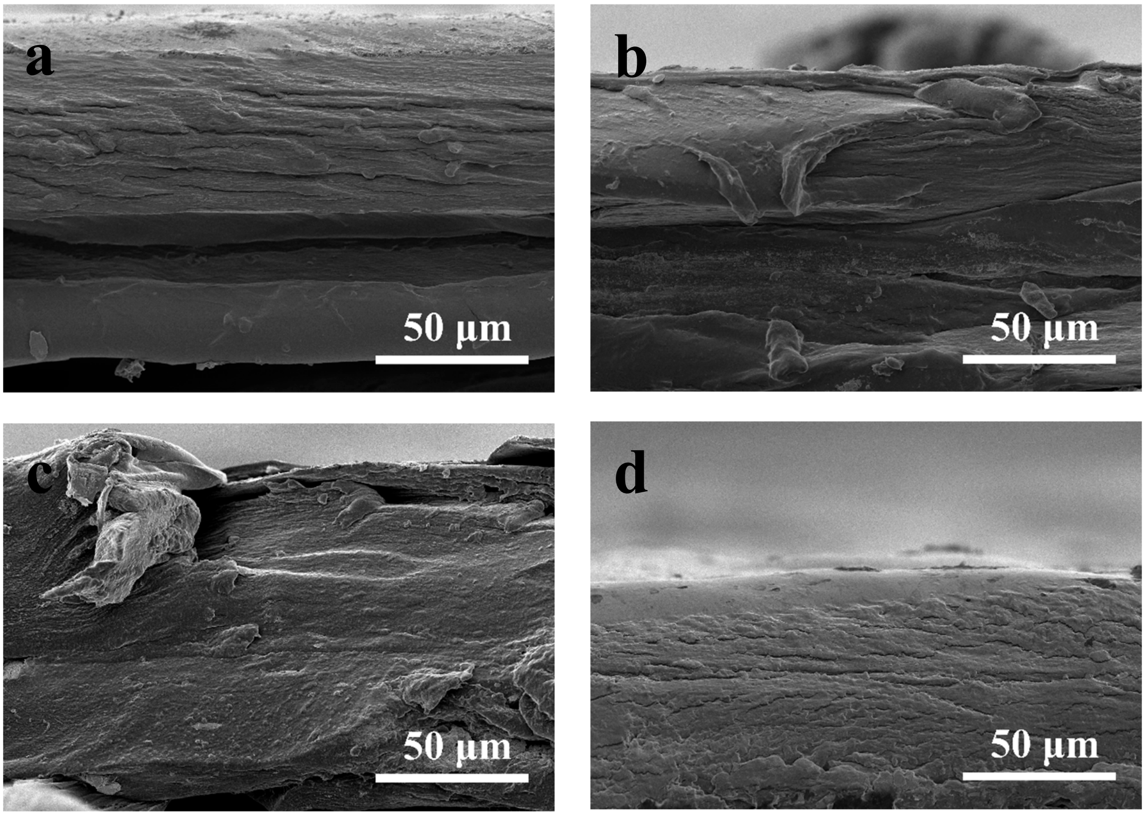

Figure S1 The SEM images of cross-sections (a) 2.5 wt %, (b) 5 wt %, (c) 10 wt %, and (d) 20 wt % MoS_2/Co_3O_4.

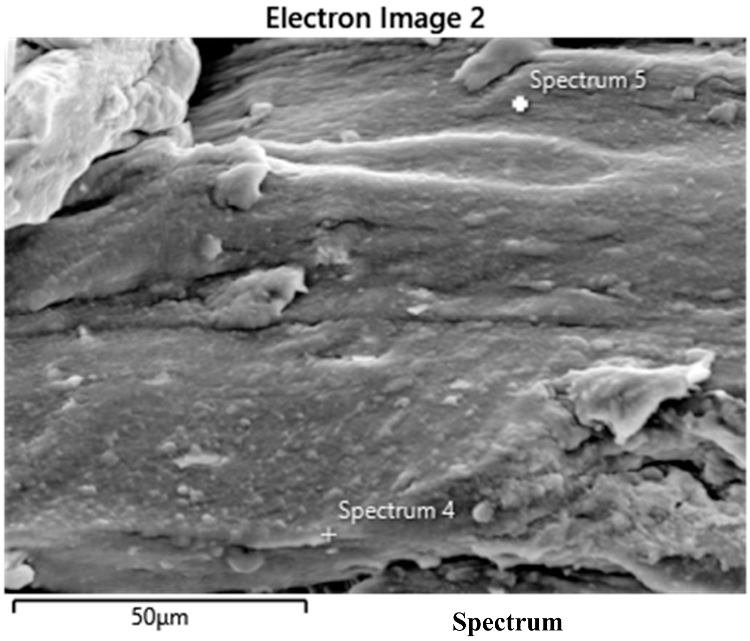

Spectrum 5

Element	Line	Wt%	At %
C	K series	68.03	76.31
O	K series	27.41	23.09
Co	K series	1.79	0.41
Au	M series	2.77	0.19
Total		100	100

Spectrum 4

Element	Line	Wt%	At %
C	K series	72.19	79.76
O	K series	23.69	19.64
S	K series	0.7	0.29
Mo	L series	1.12	0.15
Au	M series	2.3	0.15
Total		100	100

Figure S2. The EDS confirms elements of MoS_2/Co_3O_4 heterojunction.

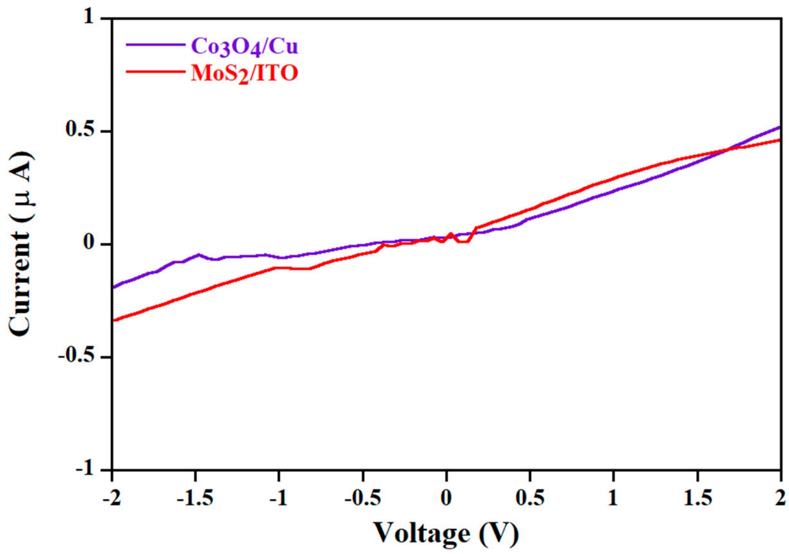

Figure S3. I-V curves of Co_3O_4/Cu and MoS_2/ITO.

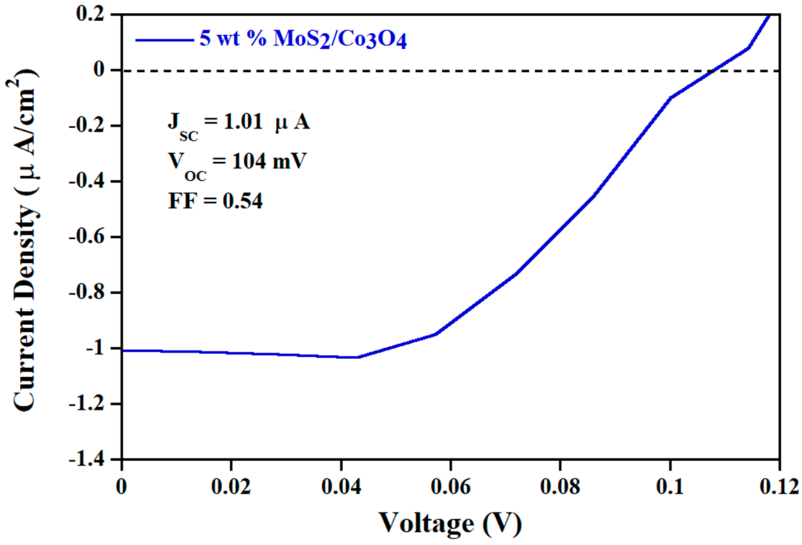

Figure S4. J-V curve of MoS_2/Co_3O_4 photodetector under the illumination of 1000 W/m^2.

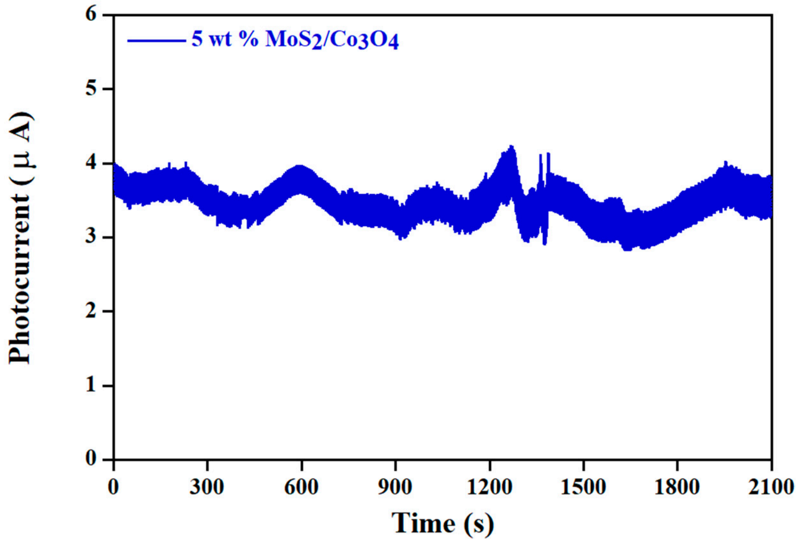

Figure S5. The stability of 5 wt % MoS_2/Co_3O_4 under light on/off 2.5 Hz for 35 mins.

S1. Hall effect via Van der Pauw method

The Hall measurement via the van der Pauw method is displayed in Figure S6. From the four values of Hall voltage, the Hall coefficient (R_H) and the carrier concentration (n) can be calculated as follows [1]:

$$R_H = \frac{\left(\frac{V_a}{I_a} - \frac{V_b}{I_b}\right) - \left(\frac{V_c}{I_c} - \frac{V_d}{I_d}\right)}{B}$$

$$n = \frac{1}{R_H e}$$

where V_a, V_b, V_c, and V_d are the changes in voltage, $I_a = I_b = I_c = I_d$ is the constant currents flowing through the sample, B is the constant magnetic field, and e is the elementary charge.

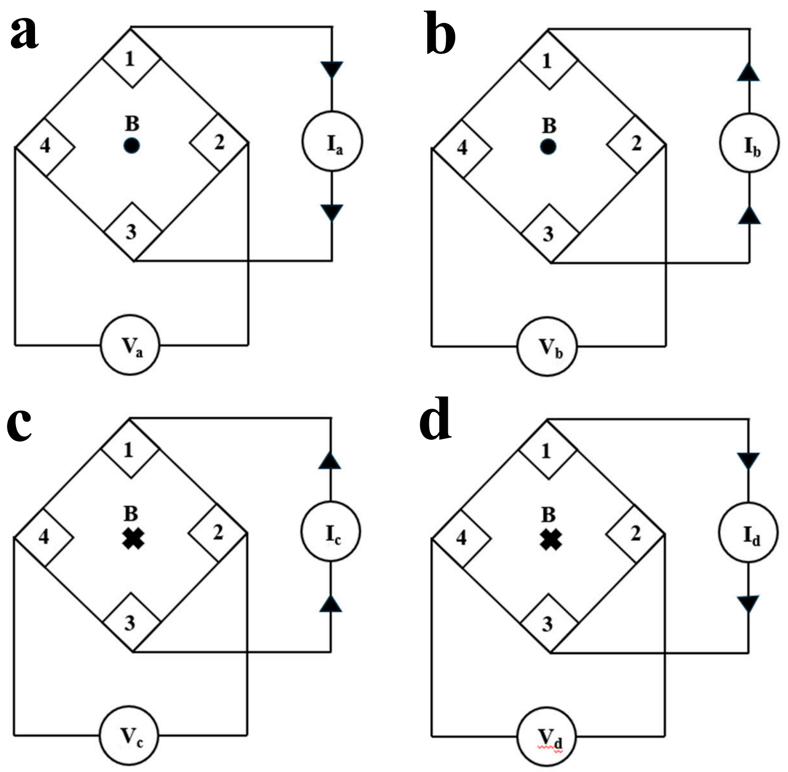

Figure S6. Diagram of Hall measurement via the van der Pauw method [1].

S2. The charge transport mechanism

By exposure of the MoS_2/Co_3O_4 heterojunction to the incident light, the electrons (e^-) in the valence band of both MoS_2 and Co_3O_4 are excited to the conduction band, leaving holes (h^+) in the valence band [2-4]. Due to the lower energy level, electrons in the conduction band of Co_3O_4 transfer to the conduction band of MoS_2 and are transported to the cathode [2-4]. Meanwhile, holes in the valence band of MoS_2 transfer to the valence band of Co_3O_4 because of the higher energy level [2-4]. After that, holes are transported to the anode. As a result of these transports, an electric current is generated [2-4].

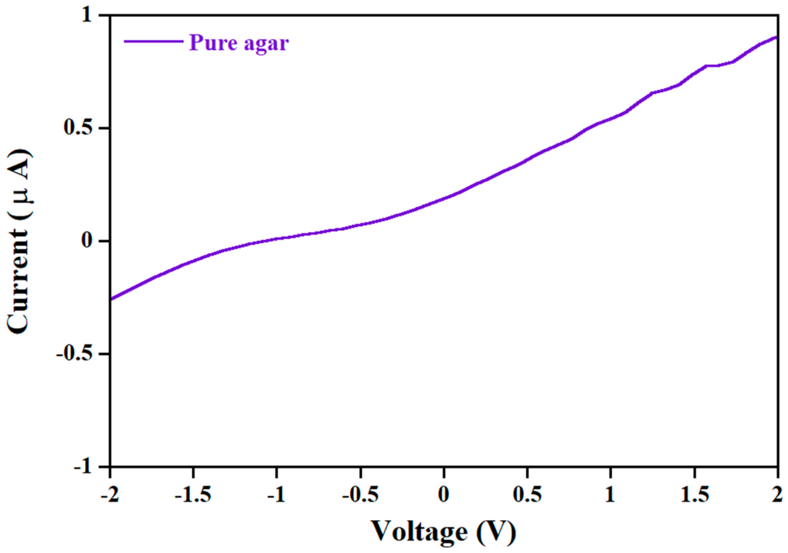

Figure S7. I-V curve of pure agar.

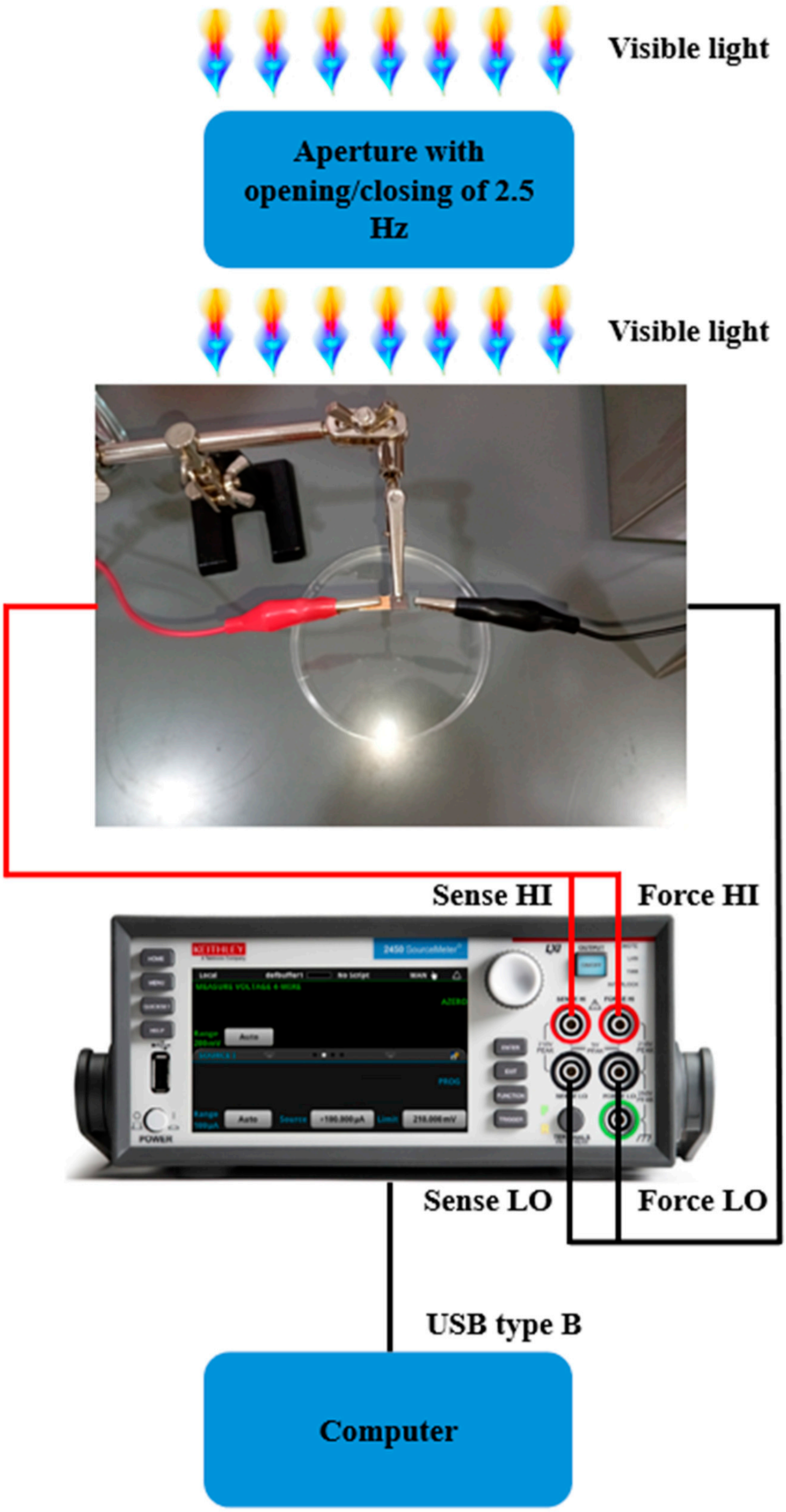

Figure S8. The setup information for the electronic measurement and the optical image of MoS_2/Co_3O_4 heterojunction as a photodetector.

References

[1] Tippo, P. Fabrication of NiO film by sparking method under a magnetic field for application in NiO/ZnO Heterojunction. The Graduate School, Chiang Mai University, Chiang Mai, 2021.

[2] Amos, S. W. Principles of Transistor Circuits, Eighth Edition: Introduction and guide to the design of amplifiers, function generators, receivers and digital circuits, 9th ed., Newnes: Jordan Hill, Oxford, England, 2000.

[3] Neamen, D. A. Semiconductor Physics And Devices: Basic Principles, fourth ed., McGraw-Hill: New York, USA, 2011.

[4] Kitai, A. Principles of Solar Cells, LEDs and Diodes: The role of the PN junction, John Wiley & Sons: New Jersey, USA, 2011.
